# Material Basis Elucidation and Quantification of Dandelion through Spectrum–Effect Relationship Study between UHPLC Fingerprint and Antioxidant Activity via Multivariate Statistical Analysis

**DOI:** 10.3390/molecules27092632

**Published:** 2022-04-20

**Authors:** Ziru Liu, Jiameng Qu, Fan Ke, Haotian Zhang, Yiwen Zhang, Qian Zhang, Qing Li, Kaishun Bi, Huarong Xu

**Affiliations:** 1 National and Local Joint Engineering Laboratory for Key Technology of Chinese Material Medica Quality Control, School of Pharmacy, Shenyang Pharmaceutical University, Shenyang 110016, China; viola18309805460@163.com (Z.L.); 18841420353@163.com (F.K.); zzzyyywen@outlook.com (Y.Z.); zhangqian@syphu.edu.cn (Q.Z.); lqyxm@hotmail.com (Q.L.); kaishunbi.syphu@gmail.com (K.B.); 2 School of Traditional Chinese Material Medica, Shenyang Pharmaceutical University, Shenyang 110016, China; qujiameng0519@163.com; 3 School of Life Science and Biopharmaceutics, Shenyang Pharmaceutical University, Shenyang 110016, China; zhanghaotian2087@163.com

**Keywords:** dandelion, antioxidant activity, spectrum–effect relationship, quantitative analysis, quality control

## Abstract

The excessive expression of reactive oxygen species is closely connected to many diseases. Considerable studies have demonstrated dandelion as well as its ingredients exhibited antioxidant activity. However, specific material basis reflecting the antioxidant activity has not been comprehensively investigated. In this study, a spectrum–effect relationship study on dandelion between fingerprinting and antioxidant activity was analyzed in detail, while a UHPLC quantification method developed and completely validated for simultaneous determination of active ingredients in dandelion. With the establishment of dandelion fingerprints of different regions, 24 common peaks were characterized. The classic FRAP method and ABTS methods were then used to detect their antioxidant activity. Partial least squares regression analysis, bivariate correlation analysis and grey correlation method were used to accomplish the spectrum–effect relationship. Eventually, the ingredients with antioxidant activity which could be considered as candidate quality markers of dandelion were discovered through spectrum–effect relationship analysis. The six compounds including caftaric acid, chlorogenic acid, caffeic acid, chicoric acid, isochlorogenic acid A, and isochlorogenic acid C were quantitatively determined. The developed UHPLC assay method was accurate, precise, and reliable. The study has elucidated the antioxidant material basis of dandelion and provided a scientific basis for the quality control of dandelion.

## 1. Introduction

Dandelion (*Taraxacum mongolicum* Hand.-Mazz) is a perennial herb in the Asteraceae family and it is also a classic traditional Chinese herbal drug. Dandelion mainly grows in temperate to subtropical regions of the Northern Hemisphere and is widely distributed in China [1,2]. According to traditional Chinese medicine (TCM) theory, dandelion is “cold” in nature and “bitter and sweet” in flavor. It returns to the “meridians” of liver and stomach, and has the effects of “clearing away heat and toxic materials”, and “removing dampness”. It is commonly used in complementary medicine for pathopyretic ulcer, jaundice, pyretic stranguria, and swelling of the eye, etc. [3]. Dandelion contains various active ingredients such as phenolic acids, flavonoids, polysaccharides, terpenoids, and sterols, which have various pharmacological effects such as anti-oxidation, anti-inflammatory, anti-tumor, hypoglycemic, hepatoprotective, and antibacterial [4,5,6,7,8].

In recent years, a large number of studies have shown that the excessive expression of reactive oxygen species in the body is closely related to many diseases, such as diabetes [9,10], aging [11,12], cancer [13,14], inflammation [8], liver disease [15], and atherosclerosis [16]. Therefore, the study of antioxidant drugs has attracted more and more attention. Meanwhile, the study of the antioxidant mechanism is of great significance for the treatment of diseases. It is reported that a variety of ingredients in dandelion have antioxidant activity [17,18,19]. However, these studies mainly focused on the antioxidant activity of monomer ingredients in dandelion, and the chemical constituents endowed with pharmacological effects are still uncertain. The study on the material basis of antioxidant efficacy of dandelion is of great significance, and this study is currently lacking.

Traditional Chinese medicine (TCM) has a long history in China. TCM is characterized by the synergistic action of multiple ingredients, multiple targets, and multiple pathways. So, in the quality control of TCM, the content quantification of a single ingredient cannot reflect its quality comprehensively [20,21]. In recent years, the quality control of TCM has made great progress. However, there are still many problems, and it is difficult to objectively evaluate and effectively control the quality of TCM [22]. The spectrum–effect relationship is generally based on the fingerprint of TCM, combined with anti-disease activity experiments, to discover the quality markers based on efficacy using chemometric statistical methods. The quality of TCM is difficult to be reflected by a single ingredient, and multiple ingredients based on efficacy can better control its quality. The spectrum–effect relationship can screen the index ingredient based on efficacy, so it can more comprehensively reflect the internal quality of TCM and optimize the deficiency caused by the lack of efficacy-based quality markers in quality control [23,24]. At the same time, it can overcome the shortcomings of chromatograms that only contain chemical characteristics and have little pharmacological information of ingredients [25]. In recent years, the spectrum–effect relationship has been widely used in the study of TCM, and has played an indispensable role in the modernization and internationalization of TCM. Moreover, it is also the primary technical means to explore the specific pharmacological material basis of TCM [26]. At present, there are few studies on quality markers based on efficacy of dandelion. Therefore, in order to better control the quality of dandelion, it is necessary to quantitatively analyze the active ingredients of dandelion.

In this study, the antioxidant activity of dandelion was used as the pharmacological index. The chromatographic fingerprints of dandelion water extracts from different origins were established. Three statistical correlation analysis methods, including partial least squares regression analysis (PLSA), bivariate analysis (BCA), and gray correlation analysis (GRA), were used to establish the spectrum–effect relationship between the fingerprints and antioxidant efficacy, and the main antioxidant ingredients in dandelion were explored. Furthermore, a series of ingredients with antioxidant activity were adopted as the characteristic ingredients in the quality control of dandelion. An UHPLC quantification method of the ingredients was successively established and completely validated, which may provide a promising basis for the quality control of dandelion.

## 2. Results and Discussion

### 2.1. UHPLC Fingerprinting

#### 2.1.1. Development of Chromatographic Method

Firstly, full-wavelength scanning was performed. The results showed that the UV absorption spectra of phenolic acids in the samples were similar, with the maximum absorption around 323 nm. Therefore, 323 nm was selected as the detection wavelength. Two separation columns, Agilent InfinityLab Poroshell 120 SB-AQ (4.6 mm × 100 mm, 2.7 μm) and Agilent InfinityLab Poroshell 120 SB-AQ (4.6 mm × 150 mm, 2.7 μm), were compared. More compounds with a clearer peak shape were detected using the latter column, so it was used for sample determination. In addition, methanol–water and acetonitrile–water systems were also investigated as mobile phases. Finally, acetonitrile–water system was selected as the mobile phase. On the one hand, the phenolic acid ingredients have certain polarity, and the elution ability of acetonitrile solution is better than that of methanol. On the other hand, the baseline was more stable when acetonitrile–water was used as the mobile phase. Moreover, 0.1% formic acid was added to both organic and aqueous phase as a modifier to improve peak tailing. Later, by adjusting the flow rate, column temperature, injection volume and gradient elution program, a variety of ingredients finally met the requirements of the fingerprint and quantitative analysis.

#### 2.1.2. Analytical Method Validation

Chicoric acid, peak No.14 with good resolution and abundant peak area, was selected as the fingerprinting reference. The results of system suitability test including the number of theoretical plates, tailing factors, resolution of chromatographic peaks, sensitivity and instrumental precision are all within acceptance. In analytical method validation, the results of precision, reproducibility, and sample stability tests were calculated based on it. The results showed that relative standard deviation (RSD) of the relative retention time and the relative peak area for each common peak was not more than 2.49% (*n* = 6), which exhibit a fine reliability of the fingerprinting method.

#### 2.1.3. Analysis of UHPLC Fingerprints and Their Similarity

**Figure 1:**
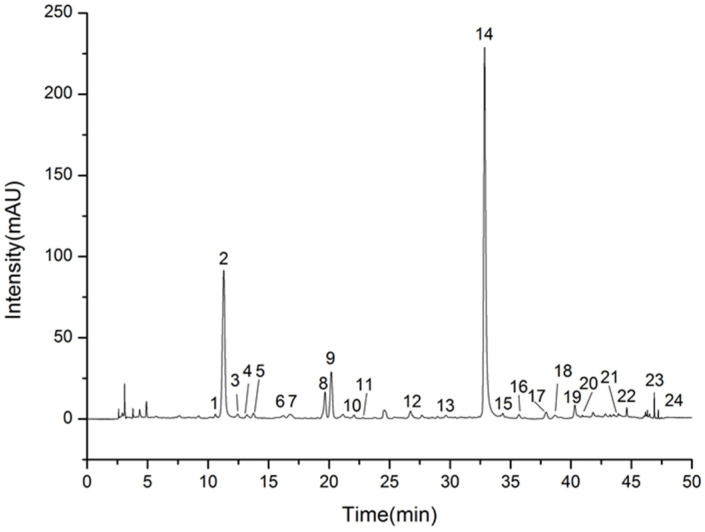
Typical LC chromatograms of sample solution.

**Figure 2:**
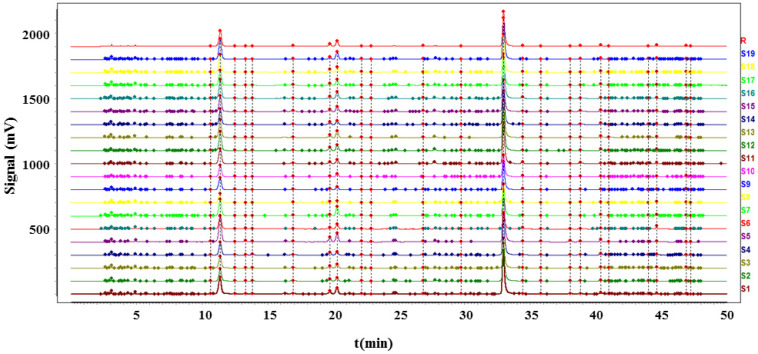
Fingerprint overlays of 19 batches of dandelion.

Nineteen batches of processed dandelion pieces from different geographical origins were used in the UHPLC fingerprinting study, and 24 common peaks were concluded, including the internal reference peak chicoric acid. The sum of common peak area was accounted for more than 95% of the total peak area, which was qualified for the fingerprinting study. Comparing with the retention time and UV absorption characteristics of the reference standards, a total of 10 chromatographic peaks were identified, including 1-neochlorogenic acid, 2-caftaric acid, 8-chlorogenic acid, 9-caffeic acid, 14-chicoric acid, 15-luteolin, 16-isochlorogenic acid B, 17-isochlorogenic acid A, 19-isochlorogenic acid C, and 22-luteoloside. The typical chromatograms of the test solution are shown in Figure 1. The similarity analysis was performed on the 19 batches of dandelion, and the similarities are shown in Table 1. Fingerprint overlays of 19 batches of dandelion are shown in Figure 2. The similarities among different batches of samples were ranged from 0.968 to 1.000.

### 2.2. Antioxidant Activity Tests

**Figure 3:**
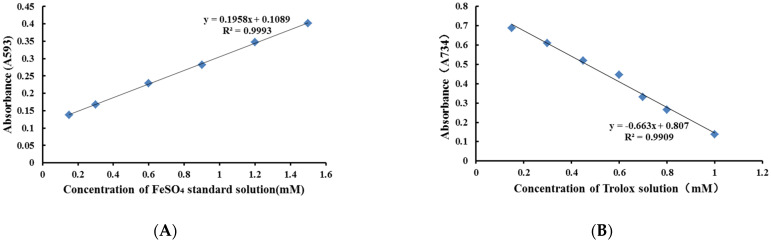
(**A**) Standard curve of FeSO_4_; (**B**) standard curve of Trolox.

The standard curve of the FeSO_4_ standard solution is shown in Figure 3A. The regression equation was *y* = 0.1958*x* + 0.1089, R^2^ = 0.9993. The total antioxidant capacity of the sample was calculated from the standard curve, and expressed as FeSO_4_ standard solution equivalents. The antioxidant activities (Ferric Reducing Ability of Plasma, FRAP) of 19 batches of dandelion are shown in Table 2.

The standard curve of the Trolox standard solution is shown in Figure 3B. The regression equation was *y* = −0.663*x* + 0.807, R^2^ = 0.9909. The total antioxidant capacity of the sample was calculated from the standard curve, and expressed as Trolox-Equivalent Antioxidant Capacity (TEAC). The antioxidant activities (2,2′-amino-di (2-ethyl-benzothiazoline sulphonic acid-6) ammonium salt, ABTS) of 19 batches of dandelion are shown in Table 2.

**Figure 4:**
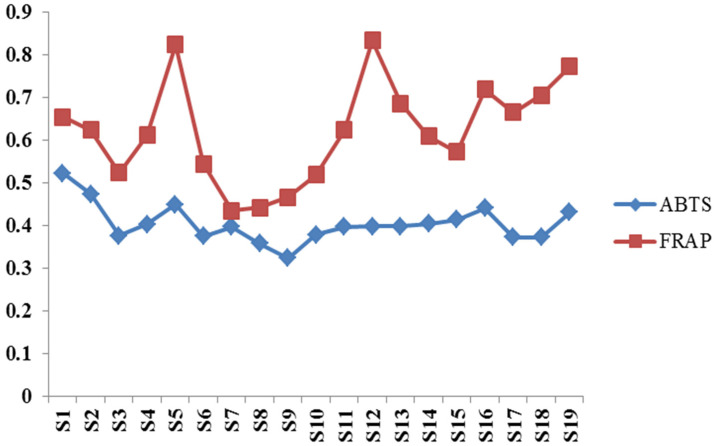
Antioxidant activities of different methods of 19 batches of dandelion.

Antioxidant activities of different methods of 19 batches of dandelion are shown in Figure 4. It can be noticed that the general trends of the two methods of the determination results for different batches of samples were in accordance, yet the orders of antioxidant activity of 19 batches of dandelion obtained by different methods remained different. This may be due to the different principles of the two antioxidant activity determination methods.

The mechanism of antioxidants can be summarized as two ways of scavenging free radicals and binding metal ions, etc., which are in accordance with the principles of ABTS and FRAP methods. For FRAP assay of total antioxidant capacity, antioxidant can reduce Fe^3+^-TPTZ under acidic conditions to produce a blue Fe^2+^-TPTZ [27], and the total antioxidant capacity in the sample can be obtained by measuring the absorbance at 593 nm subsequently. While ABTS can be oxidized to green ABTS ^+^ free radical under the action of appropriate oxidant, and the production of ABTS ^+^ free radical is inhibited in the presence of antioxidants [28]. The total antioxidant capacity of the sample can be determined and calculated by measuring the absorbance of ABTS ^+^ at 734 nm or 405 nm. The difference of principle between ABTS and FRAP assay is an important reason which lead to the difference in assay. Therefore, it is better not to use a single pharmacological indicator to evaluate the antioxidant activity, and a more comprehensive analysis should be carried out by integrating multiple characters.

### 2.3. Spectrum–Effect Relationship Analysis

#### 2.3.1. Partial Least Squares Regression Analysis (PLSR)

**Figure 5:**
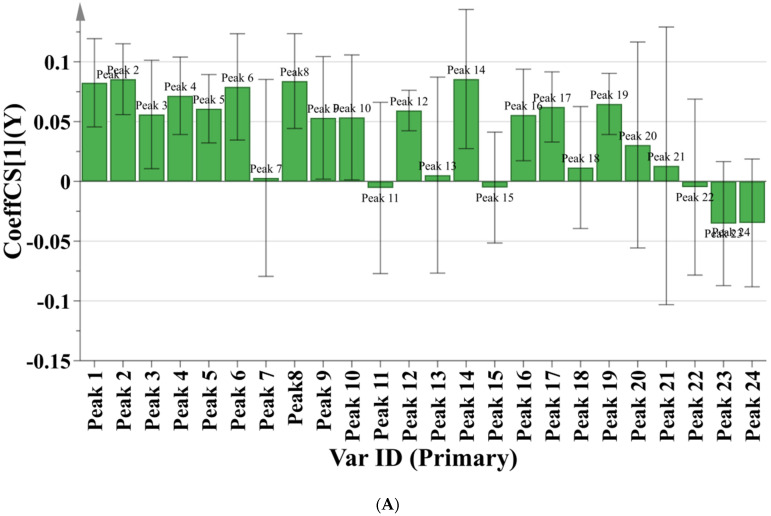

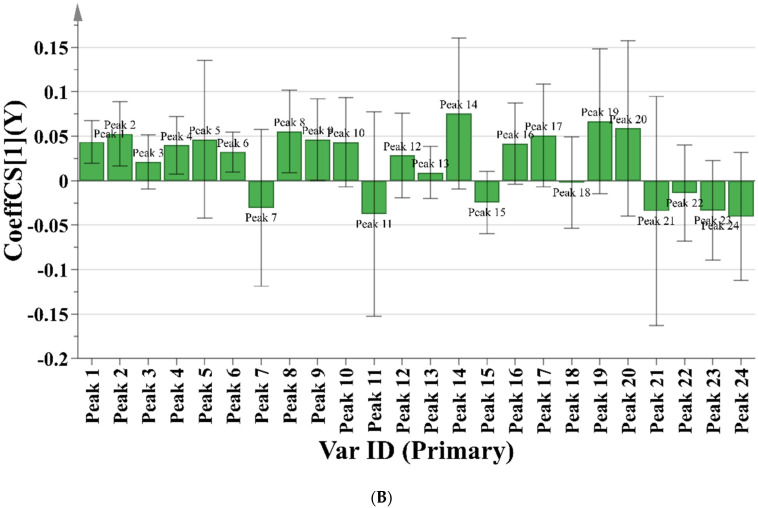
(**A**) Correlation coefficient of 24 chromatographic peaks with antioxidant capacity from FRAP assay; (**B**) Correlation coefficient of 24 chromatographic peaks with antioxidant capacity from ABTS assay.

**Figure 6:**
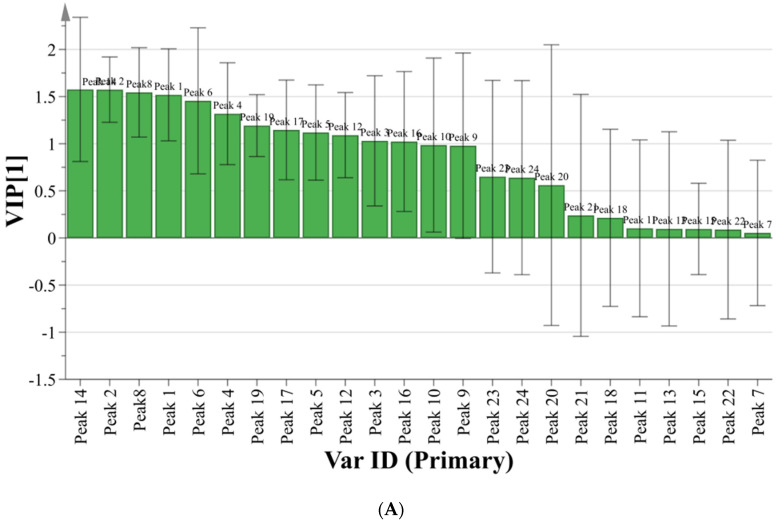

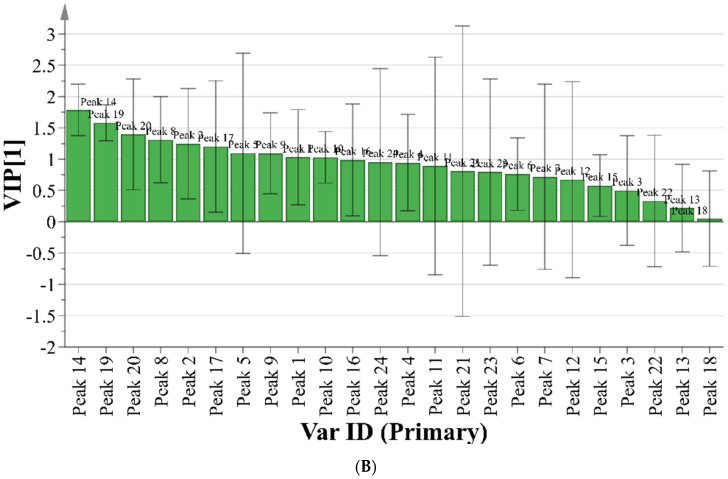
(**A**) Variable importance in projection scores (FRAP assay); (**B**) variable importance in projection scores (ABTS assay).

The FRAP assay was evaluated firstly. The correlation coefficient of 24 chromatographic peaks with antioxidant capacity is shown in Figure 5A. A positive regression coefficient stands for a positive correlation, and a negative regression coefficient stands for a negative correlation. The variable importance in projection (VIP) is shown in Figure 6A, of which VIP value > 1 indicate that the above ingredients have a significant effect on the antioxidant effect. The fitting parameter R^2^ of the model was 0.6611, the prediction parameter Q^2^ was 0.5736. Both R^2^ and Q^2^ were greater than 0.5, indicating that this model was statistically significant. In summary, the order of antioxidant activity (FRAP) of the ingredients was: chicoric acid > caftaric acid > chlorogenic acid > neochlorogenic acid > peak 6 > peak 4 > isochlorogenic acid C > isochlorogenic acid A > peak 5 > peak 12 > peak 3 > isochlorogenic acid B. The ABTS assay was analyzed in the same way and the results are shown in Figure 5B and Figure 6B. The order of antioxidant activity (ABTS) of the ingredients was: chicoric acid > isochlorogenic acid C > peak 20 > chlorogenic acid > caftaric acid > isochlorogenic acid A > peak 5 > caffeic acid > neochlorogenic acid > peak 10. The specific values of correlation coefficient and VIP value of 24 chromatographic peaks with antioxidant capacity are shown in Tables S1–S4.

#### 2.3.2. Bivariate Correlation Analysis (BCA)

Firstly, it is necessary to check whether the variables conform to normal distribution before bivariate analysis. Kolmogorov–Smirnov (K-S), Shapiro–Wilk (S-W) normality test parameters and significant values were calculated by SPSS 25.0 software. The results are shown in Table 3. When significant value is higher than 0.05, it follows normal distribution. As the number of samples was less than 100, which belonged to small sample analysis, the results were based on S-W normality test. It can be seen from the results that Peak 1, Peak 4, Peak 6, Peak 13, Peak 15, Peak 20, Peak 22, and Peak 24 did not conform to the normal distribution. Therefore, the Spearman coefficient which did not require the distribution of the original variables was selected when selecting the bivariate correlation coefficient.

Using Spearman’s correlation coefficient method, the results of Spearman′s correlation coefficient between the peak area of each common peak and antioxidant activity are shown in Table 4. The FRAP assay was evaluated firstly. Peaks 1 and 2 represented ingredients with strong correlation with antioxidant activity (r > 0.8; *p* < 0.05), and peaks 3, 4, 5, 6, 8, 9, 10, 12, and 14 represented ingredients with moderate correlation with antioxidant activity (0.5 < r < 0.8; *p* < 0.05). In summary, the order of antioxidant activity of the ingredients was: caftaric acid > neochlorogenic acid > chicoric acid > chlorogenic acid > 6 > 4 > 12 > 5 = caffeic acid > 10 > 3 > isochlorogenic acid C > isochlorogenic acid A. At the same time, the order of antioxidant activity (ABTS) of the ingredients was: chicoric acid > isochlorogenic acid C > neochlorogenic acid > chlorogenic acid > isochlorogenic acid A > caftaric acid > 5.

#### 2.3.3. Gray Correlation Analysis (GRA)

The gray correlation degrees of 24 ingredients are shown in Table 5. The closer the correlation degree is to 1, the greater the influence of the ingredient on the antioxidant activity. The results of ABTS assay were consistent with those of FRAP assay. The characteristic peaks with r > 0.8 in Table 5, combined with the identified ingredients in the fingerprint, were chicoric acid, caftaric acid, chlorogenic acid, isochlorogenic acid B, neochlorogenic acid, isochlorogenic acid C, isochlorogenic acid A, caffeic acid, and luteolin. As a result, the above substances were deduced to possess antioxidant capacity.

#### 2.3.4. Comprehensive Analysis of Spectrum–Effect Relationship

**Figure 7:**
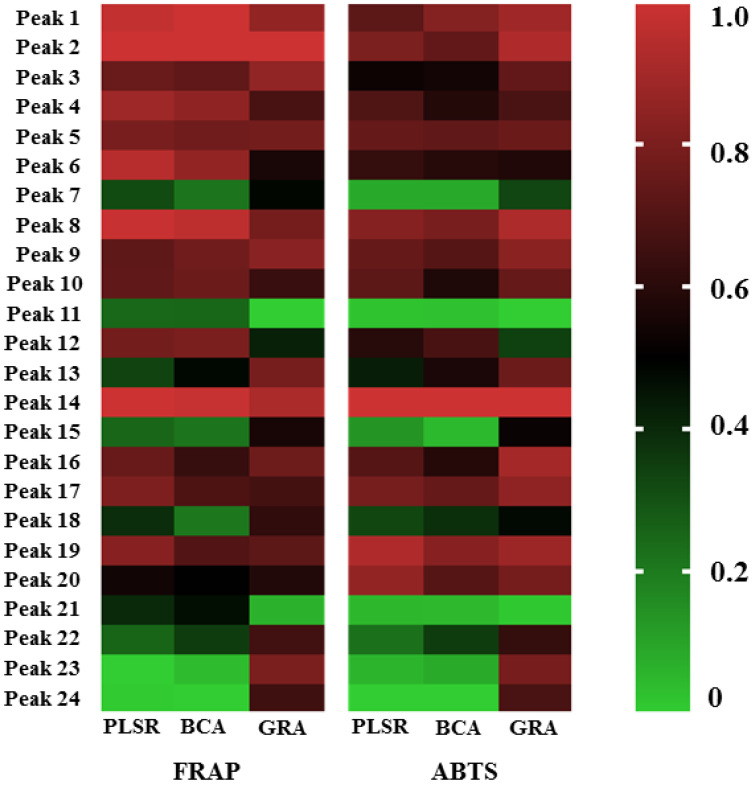
The heatmap of antioxidant correlation coefficient of 24 chromatographic peaks of different statistical methods.

**Figure 8:**
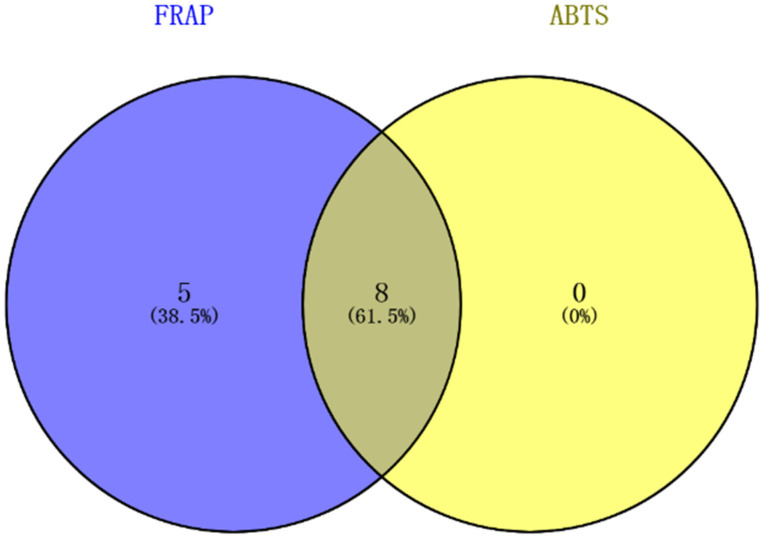
The Venn diagram of two assays.

To visualize the spectrum–effect relationship, the heatmap of antioxidant correlation coefficient of 24 chromatographic peaks of different statistical methods is shown in Figure 7. Combining the results of the above spectrum–effect relationship statistical analysis, it can be seen that the overlap of antioxidant ingredients obtained by the three methods is high, of which the results of PLSR and BCA are more consistent. Comprehensive analysis of PLSR, BCA, and GRA results showed that caftaric acid, neochlorogenic acid, chlorogenic acid, caffeic acid, chicoric acid, isochlorogenic acid A, isochlorogenic acid B, isochlorogenic acid C, peak 3, peak 4, peak 5, peak 6, and peak 12 had significant effects on antioxidant activity from FRAP assay, while chicoric acid, isochlorogenic acid C, neochlorogenic acid, chlorogenic acid, caftaric acid, caffeic acid, isochlorogenic acid A, and peak 5 had significant effects on antioxidant activity from ABTS assay. The comprehensive antioxidant material basis was concluded by taking the intersection of the above two. The Venn graph of the process is shown in Figure 8.

Finally, six of the seven common compounds with the content greater than 0.005% in dandelion were characterized as the pharmacological material basis of dandelion with antioxidant activity. The six ingredients are caftaric acid, chlorogenic acid, caffeic acid, chicoric acid, isochlorogenic acid A, and isochlorogenic acid C, which can be selected as quality markers for dandelion. The results indicated that the antioxidant efficacy of dandelion is a combination of multiple ingredients.

The principles of data analysis methods for spectrum–effect relationship are different, which may lead to different results. Each data analysis method has certain defects to some extent. BCA and GRA are methods to predict the correlation between each ingredient and the efficacy, focusing on judging the correlation between chromatographic peak and the efficacy index. However, it is difficult to describe the comprehensive contribution of each ingredient to the efficacy index. The correlation coefficients of GRA are all positive and cannot reflect whether they are positively or negatively correlated. PLSR is a method to elucidate the contribution of each ingredient to the efficacy. When the structural relationship between the independent and dependent variable is abstract, it is impossible to analyze them accurately and quantitatively [23]. A single statistical method for analysis has its own limitations and adaptability [29]. Therefore, in this study, PLSR, BCA, and GRA were used to complement each other, and the intersection analysis of the results obtained by the three methods can reflect the antioxidant material basis of dandelion more objectively and comprehensively.

### 2.4. Single Compound Verification of Antioxidant Activity

**Figure 9:**
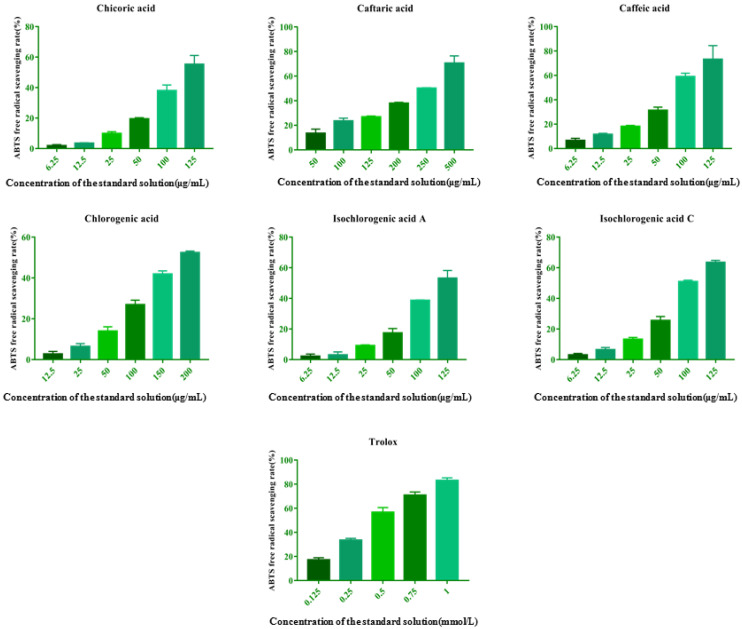
The verification results of each ingredient and positive drug (Trolox).

The verification of antioxidant activity of each ingredient, which were considered as quality maker candidates, was achieved by ABTS assay using reference substances, and the results are shown in Figure 9 and Table 6. Compared with the positive drug (Trolox), each ingredient had antioxidant activity in a concentration-dependent manner. It is proved that the results of spectrum–effect relationship were convincible.

### 2.5. Assay of Dandelion by Quantitative Analysis of Potential Antioxidant Ingredients

#### 2.5.1. Dandelion Sample Preparation

The water extract of traditional Chinese medicine is a single Chinese herbal decoction prepared by a standardized process guided by the theory of traditional Chinese medicine, with reference to modern extraction methods as well as clinical applications [30]. Based on the fact that the preparation optimized and produced by water extract is closest to the material basis of decoction in clinical practice. The extraction method of dandelion samples in this study was to prepare water extract by water extraction followed by vacuum distillation for future use. The extraction procedure was as consistent as possible with clinical medication habits and decoction methods, and prepared in strict accordance with the process parameters and research data specified in the Management Specification for Traditional Chinese Medicine Decoction Rooms in Medical Institutions. In the current research reports on dandelion water extract, the research methods have focused on single index quality evaluation [31,32], and there have been no reports on the simultaneous determination of multiple ingredients of dandelion water extract. Therefore, this study established an UHPLC-UV assay method for the six active ingredients in dandelion water extract, providing a reference for the quality control of dandelion water extract. It is worth noting that this is also the first time to study the spectrum–effect relationship of dandelion water extract and quantitatively study the effective ingredients.

#### 2.5.2. Method Validation

**Figure 10:**
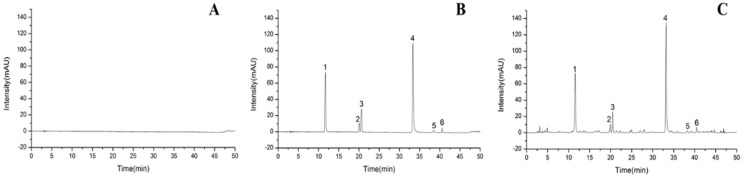
The typical LC chromatograms of blank solvent (**A**), standard solution (**B**), and sample solution (**C**). 1-caftaric acid, 2-chlorogenic acid, 3-caffeic acid, 4-chicoric acid, 5-isochlorogenic acid A, 6-isochlorogenic acid C.

The system suitability test was accomplished prior to the method validation. The resolutions between the chromatographic peaks of caftaric acid, chlorogenic acid, caffeic acid, chicoric acid, isochlorogenic acid A, and iso-chlorogenic acid C and their adjacent peaks are all greater than 1.5. All the numbers of theoretical plates are higher than 10,000, and the tailing factors are within 0.80–1.20. The signal to noise ratio of all the ingredients are greater than 10. The RSDs of the peak areas of the above six ingredients after injections of the mixed reference standard solution for six times continuously, are all not greater than 1.8%, which exhibit a good system suitability. The analytical method of quantification was validated completely. Excellent specificity of the method is well demonstrated in Figure 10. The linearity, limit of quantitation (LOQ) and limit of detection (LOD) results are shown in Table 7, and the results showed that caftaric acid, chlorogenic acid, caffeic acid, chicoric acid, isochlorogenic acid A, and isochlorogenic acid C had fine linear relationships within their respective ranges. The results of precision and accuracy were shown in Table 8 and Table 9, respectively. Recoveries and relative standard deviations (RSDs) of the peak area and content were in the acceptable range, which met the relevant requirements of quantitative analysis. The results of robustness were shown in Table 10. The RSD of each ingredient was no more than 5.00%, indicating that the method had good robustness. The results showed that the established analytical method was accurate, precise, and reliable.

#### 2.5.3. Quantification Results

The typical chromatograms of the dandelion sample solution are shown in Figure 10C. The contents of the six quality markers reflecting antioxidant activity were quantitated and the results are shown in Table 11. It can be seen from Table 11 that the contents of caftaric acid, chlorogenic acid, caffeic acid, chicoric acid, isochlorogenic acid A, and isochlorogenic acid C in 19 batches of dandelion samples were 0.1880–0.5440%, 0.01049–0.03556%, 0.01055–0.05695%, 0.1452–0.4634%, 0.001420–0.01467%, and 0.005318–0.02685%, respectively.

## 3. Materials and Methods

### 3.1. Instruments

Thermo Scientific Vanquish Horizon ultra-high performance liquid chromatography (Thermo Fisher Scientific, Waltham, MA, USA), XS105DU electronic semi-micro balance (Mettler Toledo, Greifensee, Switzerland), YP2001 electronic analytical balance (Yuyao Jinnuo Balance Instrument Co., Ltd., Yuyao, China), KQ-300E ultrasonicator (Kunshan Ultrasonic Instrument Co., Ltd., Kunshan, China), EYELA N-1100 rotary evaporator (Tokyo EYELA Instrument Co., Ltd., Tokyo, Japan), Tecan Infinite M200 Pro multifunctional microplate reader (Tecan, Switzerland).

### 3.2. Materials and Reagents

Reference standards of neochlorogenic acid, caftaric acid, chlorogenic acid, caffeic acid, chicoric acid, luteolin, isochlorogenic acid B, isochlorogenic acid A, isochlorogenic acid C, and luteoloside (purity ≥ 98%) were purchased from Chengdu Chroma Biotechnology company. Total antioxidant capacity test kit with FRAP method (Beyotime Biotechnology company, Shanghai, China, product number S0116) and total antioxidant capacity assay kit with ABTS method (Beyotime Biotechnology company, Shanghai, China, product number S0119) were used for antioxidant tests. PBS (Beijing Zhongsheng Aobang Biotechnology company, Beijing, China), methanol (HPLC-grade, Shandong Yuwang Industrial company, Shandong, China), acetonitrile (HPLC-grade, Sigma-Aldrich, St. Louis, MO, USA), formic acid (HPLC-grade, Tianjin Damao Chemical Reagent Factory) and purified water (Hangzhou Wahaha Group company, Hangzhou, China) were all met the criteria for the experiments.

Nineteen batches processed pieces of dandelion were collected from the different drug stores and were identified as *Taraxacum mongolicum* Hand.-Mazz by Professor Dong Wang from Shenyang Pharmaceutical University. The source of these processed dandelion pieces is shown in Table 12. Dandelion water extract was prepared in accordance with the process parameters specified in the Management Specification for Traditional Chinese Medicine Decoction Rooms in Medical Institutions issued by the State Administration of Traditional Chinese Medicine.

### 3.3. Development of the UHPLC Fingerprints of Dandelion

#### 3.3.1. Preparation of the Dandelion Water Extract Sample Solutions

After being soaked for 30 min, 50 g of processed dandelion pieces were reflux extracted with 600 mL purified water for 30 min. The filtrate was collected. Then another 500 mL purified water was added to the residue to continue refluxing for 20 min. The two filtrates were combined and concentrated under vacuum to 100 mL.

An accurately measured amount of 1.0 mL of each batch of dandelion water extract was transferred into a 5 mL volumetric flask, and about 3 mL methanol was added into it. Then the solution was cooled to room temperature after ultrasonication for 5 min, then diluted to volume. Finally, the sample was centrifuged at 4000× *g* in 5 min, and the supernatant was filtered by a 0.22 μm microporous filter membrane. The subsequent filtrate was stored at 4 °C for further use.

#### 3.3.2. Chromatographic Conditions

The Agilent InfinityLab Poroshell 120 SB-AQ column (4.6 mm × 150 mm, 2.7 μm) was selected as the chromatographic column and the column temperature was maintained at 35 °C The mobile phase was 0.1% formic acid in water (A) and 0.1% formic acid in acetonitrile (B). The gradient elution procedure was as follows: 0–5 min, 5–8% B; 5–13 min, 8% B; 13–17 min, 8–12% B; 17–22 min, 12% B–15% B; 22–25 min, 15–17% B; 25–29 min, 17% B–20% B; 29–35 min, 20% B; 35–40 min, 20% B–32% B; 40–43 min, 32% B–45% B; 43–45 min, 45% B–90% B; and 45–50 min, 90% B. The injection volume was 2.0 μL. The flow rate was 0.6 mL/min, and the detection wavelengths were set as 323 nm.

#### 3.3.3. Analytical Method Validation

The UHPLC system suitability test was completed prior to the method validation. The analytical method validation procedure is referred to as Technical requirements of fingerprinting study of TCM injections. Sample solutions were prepared using the dandelion pieces processed according to the method in Section 3.3.1. Instrumental precision test was evaluated by continuously injecting the samples six times under the chromatographic conditions in Section 3.3.2. The repeatability test was evaluated by six samples with the same source. The sample stability test over 24 h was assessed by injecting the same sample at different periods in a day (0, 2, 4, 8, 12, and 24 h). The retention time and peak areas were all recorded.

#### 3.3.4. Establishment of the Common Mode of Dandelion

The chromatograms of 19 batches of processed dandelion pieces were imported into the Similarity Evaluation System for Chromatographic Fingerprints of Traditional Chinese Medicine (ver. 2012, China National Software, Beijing, China) recommended by the Chinese Pharmacopoeia Commission. Then the corresponding control fingerprints were generated according to the median method after multi-point correction and data matching, and the superimposed chromatograms of 19 batches of dandelion samples were generated by the software.

### 3.4. Determination of Antioxidant Activity

#### 3.4.1. Preparation of Solutions

The FRAP working solution was prepared as follows. An amount of 1.5 mL of tripyridyltriazine (TPTZ) solution was added into 15 mL of TPTZ diluent and mixed evenly, then 1.5 mL of detection buffer was added into the solution. After mixing, it was incubated at 37 °C for further use. The sample solutions were prepared by serially diluting 19 batches of dandelion water extract prepared in Section 3.3.1 to 3 mg/mL. Meanwhile, the series standard calibration solutions were prepared as follows. An amount of 27.8 mg of FeSO_4_•7H_2_O was dissolved and diluted with purified water to a 1 mL brown volumetric flask, then the 100 mmol/L stock solution was obtained. Appropriate amounts of stock solution were diluted into 0.15, 0.3, 0.6, 0.9, 1.2, and 1.5 mmol/L. The TPTZ solution, TPTZ diluent, detection buffer, and FeSO_4_•7H_2_O were all provided by total antioxidant capacity test kit (FRAP method).

The ABTS working solution was prepared as follows. An amount of 400 μL of ABTS solution was added into 400 μL of oxidant solution and mixed evenly, then the solution was stored in the dark at room temperature for 16 h and diluted 50 times with PBS before using it. The sample solutions were prepared by serially diluting 19 batches of dandelion water extract prepared in 3.3.1 to 2.5 mg/mL. Meanwhile, the series standard calibration solutions were prepared as follows. Appropriate amounts of 10 mmol/L Trolox stock solution were diluted into 0.15, 0.30, 0.45, 0.6, 0.7, 0.8, and 1 mmol/L. The ABTS solution, oxidant solution, and Trolox stock solution (10 mM) were all provided by total antioxidant capacity assay kit with ABTS method.

#### 3.4.2. FRAP Assay

The FRAP experiment was carried out by the manufacturer′s instruction of the kit. The sample group, standard curve group and blank control group were set up. First, 180 μL of FRAP working solution was pipetted into each detection well of a 96-well microplate. Next, 5 μL of 3 mg/mL sample solution of each batch of dandelion was added to the sample well; a series of 5 μL of FeSO_4_ standard solutions were added to the standard curve well. Meanwhile, the same amount of PBS was added into the blank control well to replace the sample solution. Afterwards, they were mixed evenly by gently shaking parallel to the table and incubated at 37 °C for 5 min. Later, the absorbance was measured at 593 nm using a microplate reader. Each batch of samples was measured in triplicate and averaged. The standard curve was constructed according to the concentration and absorbance of series standard solutions.

#### 3.4.3. ABTS Assay

The ABTS experiment was carried out according to the instruction of the kit by the manufacturer. First, 200 μL of ABTS working solution was pipetted into each detection well of a 96-well microplate. Next, 10 μL of 2.5 mg/mL sample solution of each batch of dandelion was added to the sample well; a series of 10 μL of Trolox standard solutions were added to the standard curve well. Meanwhile, the same amount of PBS was added into the blank control well to replace the sample solution. Afterwards, they were mixed evenly by gently shaking parallel to the table and incubated under room temperature for 10 min. Later, the absorbance was measured at 734 nm using a microplate reader. Each batch of samples was measured in quadruplicate and averaged. The standard curve was then constructed accordingly.

### 3.5. Study of Spectrum–Effect Relationship of Dandelion

#### 3.5.1. Partial Least Squares Regression Analysis (PLSR)

Partial least squares regression analysis was performed on the measured results by SIMCA-P 14.1 software (Umetrics, Umeå, Sweden). The 24 common peak areas of 19 batches of processed dandelion pieces were set as the independent variable X and the measured results of FRAP antioxidant capacity and ABTS antioxidant capacity were set as the dependent variable Y, respectively. The regression analysis was performed using the PLSR model.

#### 3.5.2. Bivariate Correlation Analysis (BCA)

Bivariate correlation analysis was performed on the measured results using appropriate coefficient by SPSS 25.0 software (IBM, Armonk, NY, USA). The common peak areas were set as the independent variables X, and the total antioxidant capacity values were regarded as dependent variables Y.

#### 3.5.3. Gray Correlation Analysis (GRA)

Microsoft Excel software was used to analyze the gray correlation degree of the measured results. The antioxidant efficacy was set as the reference sequence. The peak area of 24 common peaks was set as the comparison sequence. Then the data were non-dimensionalized by the initial method, and the absolute difference between the comparison sequence and the reference sequence was calculated. Later, the correlation coefficient was calculated by the formula *η* (*k*) = (Δ*min + ρ*Δ*max*)/(*|Y0*(*k*) *− Yi*(*k*)*| + ρ*Δ*max*). *ρ* was usually taken as 0.5, and the average correlation coefficient of the sequence was taken as the correlation degree r.

Based on the results using the above three statistical methods, the quality markers of dandelion with antioxidant activity could be preliminarily discovered. The ABTS assay using reference substances was then used to validate the antioxidant activity of these quality markers, which could demonstrate the reliability of the multivariate analysis.

### 3.6. Assay of Dandelion by Quantitative Analysis of Potential Antioxidant Ingredients

#### 3.6.1. Preparation of the Test Solutions

Accurately measured 1.0 mL of each batch of dandelion water extract was transferred into a 10 mL volumetric flask, and about 5 mL of methanol was added. Then the solution was cooled to room temperature after ultrasonication for 5 min, then diluted to volume. Afterwards, the sample was centrifuged at 4000× *g* for 5 min, and the supernatant was filtered by a 0.22 μm microporous filter membrane. The subsequent filtrate was stored at 4 °C for further use.

Amounts of 36.70 mg of caftaric acid, 20.68 mg of chlorogenic acid, 20.40 mg of caffeic acid, 26.90 mg of chicoric acid, 19.06 mg of isochlorogenic acid A, and 20.56 mg of isochlorogenic acid C were accurately weighed. Methanol was then added to prepare a mixed reference solution containing 379.9 μg/mL of caftaric acid, 29.40 μg/mL of chlorogenic acid, 46.90 μg/mL of caffeic acid, 267.8 μg/mL of chicoric acid, 9.360 μg/mL of isochlorogenic acid A, and 11.98 μg/mL of isochlorogenic acid C.

To validate the linearity of the methodology. 0.625, 1.25, 2.5, 5, 7.5, and 10 mL of mixed reference solution was transferred into 20 mL volumetric flasks and diluted to volume with methanol. Then the series standard calibration solutions with different concentrations were prepared by shaking them.

To validate the accuracy of the methodology, 9 portions of accurately measured 1.0 mL dandelion water extract with known content were prepared. The reference substance was added at three levels of 50%, 100%, and 150% of its content, respectively. The low, medium, and high concentrations of test solution were prepared by the method under Section 3.6.1, and three samples were prepared in parallel with each concentration.

To validate the repeatability of the methodology, 0.5, 1.0, and 1.5 mL of the same batch of dandelion water extract were accurately measured. Low, medium, and high concentration standard decoction solutions were prepared according to the method under Section 3.6.1.

To validate the inter-day precision of the methodology, 1.0 mL of the same batch of dandelion water extract was accurately measured, and 3 portions of standard decoction solution were prepared with the same concentration according to the method under Section 3.6.1.

#### 3.6.2. Analytical Method Validation

The UHPLC system suitability test was compeleted first, including the number of theoretical plates, tailing factors, resolution of chromatographic peaks, sensitivity, and instrumental precision. The instrumental precision was validated by calculating the RSDs of the peak areas of the mix reference standard solution six times continously. The analytical method validation procedure referred to the guideline in the current Chinese Pharmacopeia. The specificity of the method was analyzed by comparing the chromatograms of blank solvent, reference solution and the sample solution. Next, a series of mixed reference standard solutions of different concentrations were used to evaluate the linearity. The LOD and LOQ of the analytes were also engaged to reveal the sensitivity of the method. The reference solution was diluted step by step with methanol, and the LOD was defined as signal-to-noise ratio of 3:1, and the LOQ was defined as signal-to-noise ratio of 10:1. Afterwards, the precision including repeatability (intra-day precision) and inter-day precision was performed. The intra-day precision was carried out by injecting and analyzed the samples of the low, medium, and high concentration. In addition, the inter-day precision was carried out by injecting and analyzed the samples of the same concentration for 3 consecutive days. The accuracy of the method was investigated by the recovery test. The sample stability over 24 h was assessed by injecting the sample at different time in a day (0, 2, 4, 6, 8, 12, and 24 h). The robustness is evaluated by investigating different injection volumes (±0.2 μL), detection wavelengths (±2 nm), flow rates (±0.02 mL/min), and column temperatures (±2 °C). Each condition was analyzed twice. The retention times and peak areas were all recorded.

#### 3.6.3. Sample Determination

1.0 mL of each batch of dandelion water extract was accurately measured. The test solutions were prepared according to the method under Section 3.6.1 and injected by the chromatographic conditions under Section 3.3.2.

## 4. Conclusions

In this study, a systematic study on antioxidant material basis of dandelion through spectrum–effect relationship analysis was accomplished. Bioactive ingredients of dandelion and UHPLC fingerprinting were integrated, while 19 batches of dandelion from different origins were used to successfully discover the effective antioxidant ingredients of dandelion using multivariate statistical analysis method and spectrum–effect relationship. The established spectrum–effect relationship model can evaluate the correlation between the chromatographic peaks and the pharmacological effects of the dandelion. It was concluded that caftaric acid, chlorogenic acid, caffeic acid, chicoric acid, isochlorogenic acid A, and isochlorogenic acid C might be the main ingredients of dandelion exerting antioxidant effects. A quantification method of active ingredients in dandelion was then successfully developed and completely validated. The study elucidated the antioxidant material basis of dandelion, and provided a more systematic and scientific approach for the assay of dandelion, which is a promising advance in the quality control of dandelion.

## Figures and Tables

**Table 1:** Similarity analysis results of 19 batches of dandelion.

Samples	Similarity	Samples	Similarity
S1	0.999	S11	0.998
S2	0.990	S12	1.000
S3	0.998	S13	0.999
S4	0.986	S14	0.999
S5	0.999	S15	0.999
S6	0.968	S16	0.999
S7	0.981	S17	0.996
S8	0.997	S18	1.000
S9	0.998	S19	0.999
S10	0.999		

**Table 2:** Total antioxidant capacity of 19 batches of dandelion.

Samples	FRAP (mM)	ABTS (mM)	Samples	FRAP (mM)	ABTS (mM)
S1	0.6556 ± 0.0106	0.5239 ± 0.0453	S11	0.6246 ± 0.0438	0.3975 ± 0.0327
S2	0.6251 ± 0.0049	0.4748 ± 0.0483	S12	0.8345 ± 0.0314	0.3983 ± 0.0192
S3	0.5245 ± 0.0136	0.3771 ± 0.0285	S13	0.6864 ± 0.0066	0.3991 ± 0.0182
S4	0.6120 ± 0.0259	0.4039 ± 0.0224	S14	0.6103 ± 0.0326	0.4048 ± 0.0391
S5	0.8247 ± 0.0703	0.4502 ± 0.0190	S15	0.5735 ± 0.0292	0.4151 ± 0.0336
S6	0.5455 ± 0.0161	0.3751 ± 0.0169	S16	0.7193 ± 0.0256	0.4412 ± 0.0134
S7	0.4351 ± 0.0212	0.3971 ± 0.0208	S17	0.6660 ± 0.0294	0.37383 ± 0.0261
S8	0.4430 ± 0.0099	0.3580 ± 0.0339	S18	0.7063 ± 0.0351	0.37379 ± 0.0451
S9	0.4677 ± 0.0253	0.3241 ± 0.0342	S19	0.7729 ± 0.0421	0.4320 ± 0.0176
S10	0.5194 ± 0.0049	0.3789 ± 0.0356			

**Table 3:** Results of normality test.

No.	Kolmogorov–Smirnov ^a^		Shapiro–Wilk	
	Result	Sig.	Result	Sig.
Peak 1	0.166	0.176	0.878	0.020
Peak 2	0.124	0.200 *	0.942	0.286
Peak 3	0.183	0.095	0.940	0.263
Peak 4	0.134	0.200 *	0.876	0.018
Peak 5	0.097	0.200 *	0.970	0.770
Peak 6	0.198	0.047	0.859	0.010
Peak 7	0.141	0.200 *	0.966	0.692
Peak 8	0.169	0.159	0.916	0.096
Peak 9	0.119	0.200 *	0.967	0.723
Peak 10	0.132	0.200 *	0.973	0.838
Peak 11	0.107	0.200 *	0.954	0.454
Peak 12	0.148	0.200 *	0.970	0.780
Peak 13	0.239	0.006	0.861	0.010
Peak 14	0.116	0.200 *	0.975	0.865
Peak 15	0.200	0.043	0.896	0.041
Peak 16	0.153	0.200 *	0.952	0.431
Peak 17	0.135	0.200 *	0.939	0.253
Peak 18	0.121	0.200 *	0.976	0.881
Peak 19	0.136	0.200 *	0.971	0.798
Peak 20	0.265	0.001	0.839	0.005
Peak 21	0.163	0.197	0.940	0.263
Peak 22	0.219	0.017	0.721	0.000
Peak 23	0.148	0.200 *	0.917	0.098
Peak 24	0.187	0.078	0.819	0.002
FRAP	0.087	0.200 *	0.969	0.760
ABTS	0.188	0.077	0.932	0.188

^a^ Lilliefors Significance Correction. * This is a significant lower limit of true.

**Table 4:** Spearman correlation coefficient between variables and antioxidant activity data.

No.	FRAP	ABTS	No.	FRAP	ABTS
Peak 1	0.805 **	0.547 *	Peak 13	0.263	0.279
Peak 2	0.809 **	0.460 *	Peak 14	0.789 **	0.735 **
Peak 3	0.528 *	0.258	Peak 15	−0.018	−0.263
Peak 4	0.649 **	0.302	Peak 16	0.423	0.305
Peak 5	0.568 *	0.458 *	Peak 17	0.479 *	0.467 *
Peak 6	0.658 **	0.307	Peak 18	−0.025	0.089
Peak 7	−0.019	−0.221	Peak 19	0.491 **	0.554 *
Peak 8	0.767 **	0.516 *	Peak 20	0.277	0.430
Peak 9	0.568 *	0.430	Peak 21	0.244	−0.260
Peak 10	0.558 *	0.288	Peak 22	0.125	0.056
Peak 11	0.018	−0.282	Peak 23	−0.204	−0.226
Peak 12	0.595 **	0.396	Peak 24	−0.246	−0.312

“**” indicates the correlation is significant at the 0.01 level; “*”—indicates the correlation is significant at the 0.05 level.

**Table 5:** Gray relational analysis results between variables and antioxidant activity data.

No.	R (FRAP)	R (ABTS)	No.	R (FRAP)	R (ABTS)
Peak 1	0.898	0.942	Peak 13	0.861	0.848
Peak 2	0.909	0.919	Peak 14	0.930	0.846
Peak 3	0.854	0.902	Peak 15	0.7927	0.845
Peak 4	0.838	0.901	Peak 16	0.901	0.841
Peak 5	0.861	0.896	Peak 17	0.887	0.836
Peak 6	0.808	0.886	Peak 18	0.7794	0.825
Peak 7	0.7366	0.883	Peak 19	0.895	0.819
Peak 8	0.907	0.882	Peak 20	0.868	0.8187
Peak 9	0.883	0.881	Peak 21	0.6447	0.7978
Peak 10	0.857	0.877	Peak 22	0.822	0.7798
Peak 11	0.6413	0.865	Peak 23	0.869	0.6808
Peak 12	0.7402	0.851	Peak 24	0.838	0.6624

**Table 6:** IC_50_ values (μg/mL) of of each ingredient and positive drug (Trolox).

Analytes	IC_50_ (μg/mL)
Caftaric acid	263.3
Chlorogenic acid	190.9
Caffeic acid	73.78
Chicoric acid	121.2
Isochlorogenic acid A	123.7
Isochlorogenic acid C	93.6
Trolox	99.1

**Table 7:** Linearity, LOQ, and LOD of the quantification method validation.

Analytes	Linearity			LOD (μg/mL)	LOQ (μg/mL)
	Range (μg/mL)	Equation	*R* ^2^		
Caftaric acid	45.88–734.0	*y* = 5.875*x* − 14.91	0.9990	1.311	2.263
Chlorogenic acid	3.671–58.73	*y* = 10.64*x* − 8.36	0.9994	1.477	2.954
Caffeic acid	5.865–93.8	*y* = 18.98*x* − 7.389	0.9998	1.457	2.914
Chicoric acid	33.63–538.0	*y* = 13.329*x* − 7.429	0.9993	1.429	2.857
Isochlorogenic acid A	1.191–19.06	*y* = 11.91*x* − 4.120	0.9998	1.361	2.723
Isochlorogenic acid C	1.491–23.85	*y* = 10.89x − 6.476	0.9997	1.469	2.937

**Table 8:** Precisions of the quantification method validation.

Analytes	Intra-Day (*n* = 9)	Inter-Day (*n* = 9)	Stability (*n* = 7)
	RSD (%)	RSD (%)	RSD (%)
Caftaric acid	2.99	0.70	0.98
Chlorogenic acid	1.51	0.97	1.10
Caffeic acid	1.20	0.87	1.14
Chicoric acid	1.79	0.69	1.05
Isochlorogenic acid A	3.36	0.93	1.56
Isochlorogenic acid C	2.14	0.74	1.21

**Table 9:** Recoveries of the quantification method validation (*n* = 9).

Analytes	Original (μg)	Spiked (μg)	Found (μg)	Recovery (%)	RSD (%)
Caftaric acid	1956	1005	2868	90.8	0.5
		1956	3815	95.1	1.1
		2934	4723	94.3	0.2
Chlorogenic acid	153.6	76.52	219.4	86.0	0.4
		154.1	285.6	85.7	0.2
		230.6	364.2	91.4	0.2
Caffeic acid	211.0	105.1	302.4	87.0	0.5
		211.1	395.1	87.2	0.1
		316.2	518.2	97.2	0.3
Chicoric acid	1529	760	2229	92.1	1.0
		1530	2994	95.8	0.4
		2293	3828	100.2	0.6
Isochlorogenic acid A	42.38	19.06	59.34	89.0	1.9
		41.93	80.22	90.3	0.8
		63.85	95.61	83.4	0.5
Isochlorogenic acid C	63.79	31.87	96.58	102.9	0.3
		63.74	119.9	88.0	1.6
		95.60	162.9	103.6	1.7

**Table 10:** Robustness of the quantification method validation (*n* = 6).

Analytes	Injection Volume (±0.2 μL, *n* = 6)	Detection Wavelength (±2 nm, *n* = 6)	Flow Rate (±0.02 mL/min, *n* = 6)	Column Temperature (±2 °C, *n* = 6)
	RSD (%)	RSD (%)	RSD (%)	RSD (%)
Caftaric acid	0.93	2.72	1.06	1.59
Chlorogenic acid	1.11	2.84	0.81	0.53
Caffeic acid	0.99	2.94	0.78	0.55
Chicoric acid	0.79	2.32	1.26	0.95
Isochlorogenic acid A	1.47	2.92	4.63	4.21
Isochlorogenic acid C	4.01	4.97	4.97	3.57

**Table 11:** The content of 6 ingredients in the 19 batches of dandelion water extract (%).

Batch No.	Caftaric Acid	Chlorogenic Acid	Caffeic Acid	Chicoric Acid	Isochlorogenic Acid A	Isochloro-Genic Acid C
S1	0.3477	0.03262	0.03590	0.3505	0.00898	0.01670
S2	0.2665	0.02395	0.02205	0.3319	0.003640	0.01232
S3	0.2586	0.02420	0.02363	0.2551	0.006327	0.01271
S4	0.2446	0.02552	0.01872	0.3245	0.005602	0.01517
S5	0.5440	0.05649	0.05507	0.4634	0.01467	0.02685
S6	0.2569	0.01524	0.02944	0.1452	0.003576	0.007548
S7	0.3494	0.01766	0.05695	0.2182	0.006782	0.01527
S8	0.1880	0.01656	0.01055	0.1563	0.00948	0.007377
S9	0.2588	0.01496	0.01815	0.2182	0.002433	0.007565
S10	0.2346	0.01049	0.01867	0.1839	0.001420	0.005318
S11	0.3074	0.01982	0.02314	0.3102	0.002905	0.00858
S12	0.3763	0.03556	0.03408	0.3265	0.00873	0.01664
S13	0.4000	0.03060	0.04232	0.3071	0.00896	0.01924
S14	0.2875	0.02566	0.02477	0.2623	0.006437	0.01400
S15	0.2590	0.02125	0.02641	0.2075	0.006813	0.01500
S16	0.3893	0.02328	0.03983	0.3543	0.004142	0.00998
S17	0.2700	0.01787	0.03060	0.1968	0.005435	0.01207
S18	0.2954	0.02912	0.02821	0.2502	0.005783	0.01185
S19	0.2906	0.02030	0.03558	0.2383	0.004094	0.007988

**Table 12:** Sample information of dandelion.

Sample No.	Batch No.	Origin
S1	1908005	Henan
S2	20191117	Henan
S3	190801	Henan
S4	C3312001001	Henan
S5	20201001	Henan
S6	201110	Shanxi
S7	191101	Shanxi
S8	190701	Shanxi
S9	180804	Gansu
S10	180805	Gansu
S11	191201	Gansu
S12	2007008	Hebei
S13	2006067	Hebei
S14	2003002	Hebei
S15	201101	Anhui
S16	200301	Anhui
S17	200401309	Hubei
S18	D20100103	Hubei
S19	D20030103	Hubei

## Data Availability

Samples of the compounds are available from the authors.

## Supplementary Materials

The following supporting information can be downloaded at: https://www.mdpi.com/article/10.3390/molecules27092632/s1, Table S1: Correlation coefficient of 24 chromatographic peaks with antioxidant capacity of PLSR (FRAP); Table S2: VIP value of 24 chromatographic peaks with antioxidant capacity of PLSR (FRAP); Table S3: Correlation coefficient of 24 chromatographic peaks with antioxidant capacity of PLSR (ABTS); Table S4: VIP value of 24 chromatographic peaks with antioxidant capacity of PLSR (ABTS).
