# Supplementary material for: Material Basis Elucidation and Quantification of Dandelion through Spectrum–Effect Relationship Study between UHPLC Fingerprint and Antioxidant Activity via Multivariate Statistical Analysis
Source: Molecules. 2022 Apr 20;27(9):2632. doi: 10.3390/molecules27092632 (PMC9101216; doi:10.3390/molecules27092632)
Supplement: Supplementary file 1 [file molecules-27-02632-s001.zip › molecules-1655317-supplementary.pdf]

Supplementary Materials

Material Basis Elucidation and Quantification of Dandelion through Spectrum–Effect Relationship Study between UHPLC Fingerprint and Antioxidant Activity via Multivariate Statistical Analysis

Ziru Liu [1], Jiameng Qu [2], Fan Ke [1], Haotian Zhang [3], Yiwen Zhang [1], Qian Zhang [1], Qing Li [1], Kaishun Bi [1] and Huarong Xu [1,*]

[1] National and Local Joint Engineering Laboratory for Key Technology of Chinese Material Medica Quality Control, School of Pharmacy, Shenyang Pharmaceutical University, Shenyang 110016. China; viola18309805460@163.com (Z.L.); 18841420353@163.com (F.K.); zzzyyywen@outlook.com (Y.Z.); zhangqian@syphu.edu.cn (Q.Z.); lqyxm@hotmail.com (Q.L.); kaishunbi.syphu@gmail.com (K.B.)
[2] School of Traditional Chinese Material Medica, Shenyang Pharmaceutical University, Shenyang 110016, China; qujiameng0519@163.com
[3] School of Life Science and Biopharmaceutics, Shenyang Pharmaceutical University, Shenyang 110016, China; zhanghaotian2087@163.com
* Correspondence: xuh@syphu.edu.cn; Tel.: +86-24-43-520-588

TABLE S1. Correlation coefficient of 24 chromatographic peaks with antioxidant capacity of PLSR(FRAP)

No.	correlation	No.	correlation
Peak 1	0.082468	Peak 13	0.005224
Peak 2	0.085474	Peak 14	0.08559
Peak 3	0.055967	Peak 15	-0.00518
Peak 4	0.071585	Peak 16	0.05557
Peak 5	0.060765	Peak 17	0.062259
Peak 6	0.079032	Peak 18	0.011582
Peak 7	0.002903	Peak 19	0.064768
Peak 8	0.083874	Peak 20	0.03043
Peak 9	0.053145	Peak 21	0.013008
Peak 10	0.053513	Peak 22	-0.00481
Peak 11	-0.00553	Peak 23	-0.03533
Peak 12	0.059264	Peak 24	-0.03475

TABLE S2. VIP value of 24 chromatographic peaks with antioxidant capacity of PLSR (FRAP)

No.	VIP	No.	VIP
Peak 14	1.57558	Peak 10	0.9851
Peak 2	1.57345	Peak 9	0.978312
Peak 8	1.544	Peak 23	0.650324
Peak 1	1.51812	Peak 24	0.639667
Peak 6	1.45486	Peak 20	0.560168
Peak 4	1.31777	Peak 21	0.239459
Peak 19	1.19229	Peak 18	0.21321
Peak 17	1.1461	Peak 11	0.101803
Peak 5	1.1186	Peak 13	0.096163
Peak 12	1.09096	Peak 15	0.0953
Peak 3	1.03026	Peak 22	0.088537
Peak 16	1.02297	Peak 7	0.053438

TABLE S3. Correlation coefficient of 24 chromatographic peaks with antioxidant capacity of PLSR (ABTS)

No.	correlation	No.	correlation
Peak 1	0.043545	Peak 13	0.009201
Peak 2	0.052681	Peak 14	0.07557
Peak 3	0.021075	Peak 15	-0.02444
Peak 4	0.039848	Peak 16	0.041669
Peak 5	0.046334	Peak 17	0.050885
Peak 6	0.032191	Peak 18	-0.00216
Peak 7	-0.03036	Peak 19	0.06685
Peak 8	0.055384	Peak 20	0.058953
Peak 9	0.046202	Peak 21	-0.03409
Peak 10	0.043483	Peak 22	-0.01404
Peak 11	-0.03762	Peak 23	-0.03356
Peak 12	0.028464	Peak 24	-0.04028

TABLE S4. VIP value of 24 chromatographic peaks with antioxidant capacity of PLSR (ABTS)

No.	VIP	No.	VIP
Peak 14	1.78735	Peak 4	0.942484
Peak 19	1.58112	Peak 11	0.889849
Peak 20	1.39433	Peak 21	0.806381
Peak 8	1.30993	Peak 23	0.79382
Peak 2	1.246	Peak 6	0.761371
Peak 17	1.20353	Peak 7	0.718094
Peak 5	1.09587	Peak 12	0.673218
Peak 9	1.09277	Peak 15	0.57805
Peak 1	1.02992	Peak 3	0.498459
Peak 10	1.02844	Peak 22	0.332137
Peak 16	0.985551	Peak 13	0.217625
Peak 24	0.952672	Peak 18	0.051104
